# Arbuscular Mycorrhizal Fungi and Soil Quality Indicators in *Eucalyptus* genotypes With Different Drought Tolerance Levels

**DOI:** 10.3389/ffunb.2022.913570

**Published:** 2022-06-20

**Authors:** Bruna Andreia de Bacco Lopes, Antonio Marcos Miranda Silva, Maiele Cintra Santana, Henrique Petry Feiler, Arthur Prudêncio de Araújo Pereira, Marcos Ferreira Teixeira, Victor Lucas Vieira Prudêncio de Araújo, Patrícia Andressa de Ávila, José Leonardo de Moraes Gonçalves, Siobhan Staunton, Elke Jurandy Bran Nogueira Cardoso

**Affiliations:** ^1^ Department of Soil Science, “Luiz de Queiroz” College of Agriculture, University of São Paulo, São Paulo, Brazil; ^2^ Soil Microbiology Laboratory, Soil Science Department, Federal University of Ceará, Ceará, Brazil; ^3^ Department of Forest Sciences, “Luiz de Queiroz” College of Agriculture, University of São Paulo, São Paulo, Brazil; ^4^ Eco&Sols, INRAE-IRD-Cirad-SupAgro, Montpellier, France

**Keywords:** mycorrhiza, symbiosis, plant-fungal interaction, soil microbes, water stress, soil health assessment

## Abstract

Silviculture has great importance worldwide, and the use of *Eucalyptus* species, which account for 75% of the local planted forest in Brazil, is one of the factors that contributes to the success of this activity in the country. Despite its adaptability, the yield of *Eucalyptus* is often affected by climate change, particularly water deficiency. Plants have developed strategies to mitigate water stress, for example, through their association with mycorrhizal fungi. The genus *Eucalyptus*, particularly in the plant domain, establishes symbioses with arbuscular mycorrhizal fungi (AMF) and ectomycorrhizal fungi (ECMF). The influence of *Eucalyptus* species on AMF and soil quality indicators is not well understood. Our aim was to conduct a preliminary evaluation of the various responses of soil AMF communities and soil nutrient dynamics in the presence of *Eucalyptus* species with different degrees of drought tolerance. A field experiment was established containing six *Eucalyptus* species, *E. brassiana*, *E. camaldulensis*, *E. citriodora*, *E. cloeziana*, *E. grandis*, and *E. urophylla*, all of which were planted in large plots. Soil and root samples were taken when the plants were 1.7 and 2.2 years old. We found that *Eucalyptus* species with low (*E. grandis* and *E. urophylla*) and intermediate drought tolerance (*E. citriodora* and *E. cloeziana*) showed stronger correlations with the AMF community than *Eucalyptus* species with high drought tolerance (*E. brassiana* and *E. camaldulensis*). Differences were also found between *Eucalyptus* species for AMF spore numbers and root colonization percentages, which was most evident for *E. urophylla*. The microbiological attributes found to be most responsive to *Eucalyptus* species were soil enzyme activities, AMF spore numbers, root colonization percentages, and fungal abundance. Soil organic carbon, phosphorus, potassium, zinc, copper, and iron were the main chemical drivers related to the soil AMF community structure in the presence of *E. brassiana*.

## 1 Introduction

The genus *Eucalyptus* has growing importance worldwide. Brazil is one of its major producers, with *Eucalyptus* species accounting for 75% of Brazilian planted forests (Ibá, 2020). Most forest products come from *Eucalyptus* trees, and this predominance is due to its fast growth, good adaptation to different soils and climates, and economic potential. However, *Eucalyptus* suffers from severe growth limitations under water deficiency, especially in the tropics (Embrapa, 2014). In this context, one of the main challenges facing this species is to maintain high yields despite climate adversities, which requires an understanding of the changes in soil microbial communities, as they play a key role in the functioning of soil forests (Meyer et al., 2020; Steidinger et al., 2020).

Fungi are among the most important components of the soil microbial community in forest plantations, providing fundamental ecosystem services and promoting forest health (Duffy et al., 2017). Arbuscular mycorrhizal fungi (AMF) and ectomycorrhizal fungi (ECMF) are protagonists of a sustainable forest, working as extensions of the roots, helping in the uptake of water and nutrients and functioning in other ecosystem services, such as pathogen control (Bowles et al., 2016; Cardoso and Andreote, 2016). The genus *Eucalyptus* establishes mycorrhizal associations with both kinds of fungi and is an important element in the so-called bioeconomy, for which knowledge about underground interactions has to be incorporated increasingly into forestry research, in particular to elucidate how mycorrhizal symbiosis drives soil community biology (Meyer et al., 2020; Tedersoo et al., 2020; Pereira et al., 2021; Che et al., 2022; Wang et al., 2022). AMF predominate during the first two years of *Eucalyptus* seedling growth, whereas ECMF becomes more abundant in older trees (Santos et al., 2001).

All plants have or can develop physiological mechanisms that enable them to avoid low water potentials (avoidance) or tolerate dehydration (tolerance) (Brunner et al., 2015). Another strategy often adopted by plants to cope with dry periods is the association between roots and soil microorganisms. Colonization by mycorrhizal fungi might lead to distinct ecological relationships with trees, such as symbiosis, competition, commensalism, synergism, and antagonism, thereby affecting the colonization process of each tree species (Chilvers et al., 1987; Tedersoo et al., 2020).

*Eucalyptus* species can be classified according to their level of drought tolerance (Gonçalves et al., 2017). Several studies have demonstrated differences in soil fungal and bacterial communities depending on the vegetation due to plant species-specific variations in root traits and soil properties (Carnovale et al., 2019; Fu et al., 2020). However, there is still a paucity of information on how different tree species interact with the arbuscular mycorrhizal community and soil quality indicators. A better understanding of the responses of AMF communities in *Eucalyptus* plantations is required, especially in the context of increasing climate change (Booth, 2013). Therefore, comparisons of the AMF community and soil nutrient dynamics in association with different *Eucalyptus* species may elucidate plant-soil feedback mechanisms. The present study is an exploratory project to gain a better understanding of the influence of *Eucalyptus* species on soil fungal community traits, especially on arbuscular mycorrhizal symbiosis, as well as their interactions with soil chemical and microbiological attributes under field conditions.

## 2 Material and Methods

### 2.1 Site Description and Experimental Design

The field assay was set up in March 2016 at the Experimental Station of Forest Sciences (23°02’S, 48°38’W, 830 m above sea level) of the “Luiz de Queiroz” College of Agriculture (University of São Paulo, Brazil) in an area previously occupied by a clonal *Eucalyptus* plantation. The climate is Cfa (humid subtropical) according to Köppen’s classification. The mean annual temperature and rainfall were 19°C and 1,350 mm, respectively. The soil was classified as Typic Hapludox (Soil Survey Staff, 2014), corresponding to Latossolo Vermelho-Amarelo (red-yellow latosol), with a loamy texture from the Brazilian Soil Classification Staff (Santos et al., 2018).

**Figure 1:**
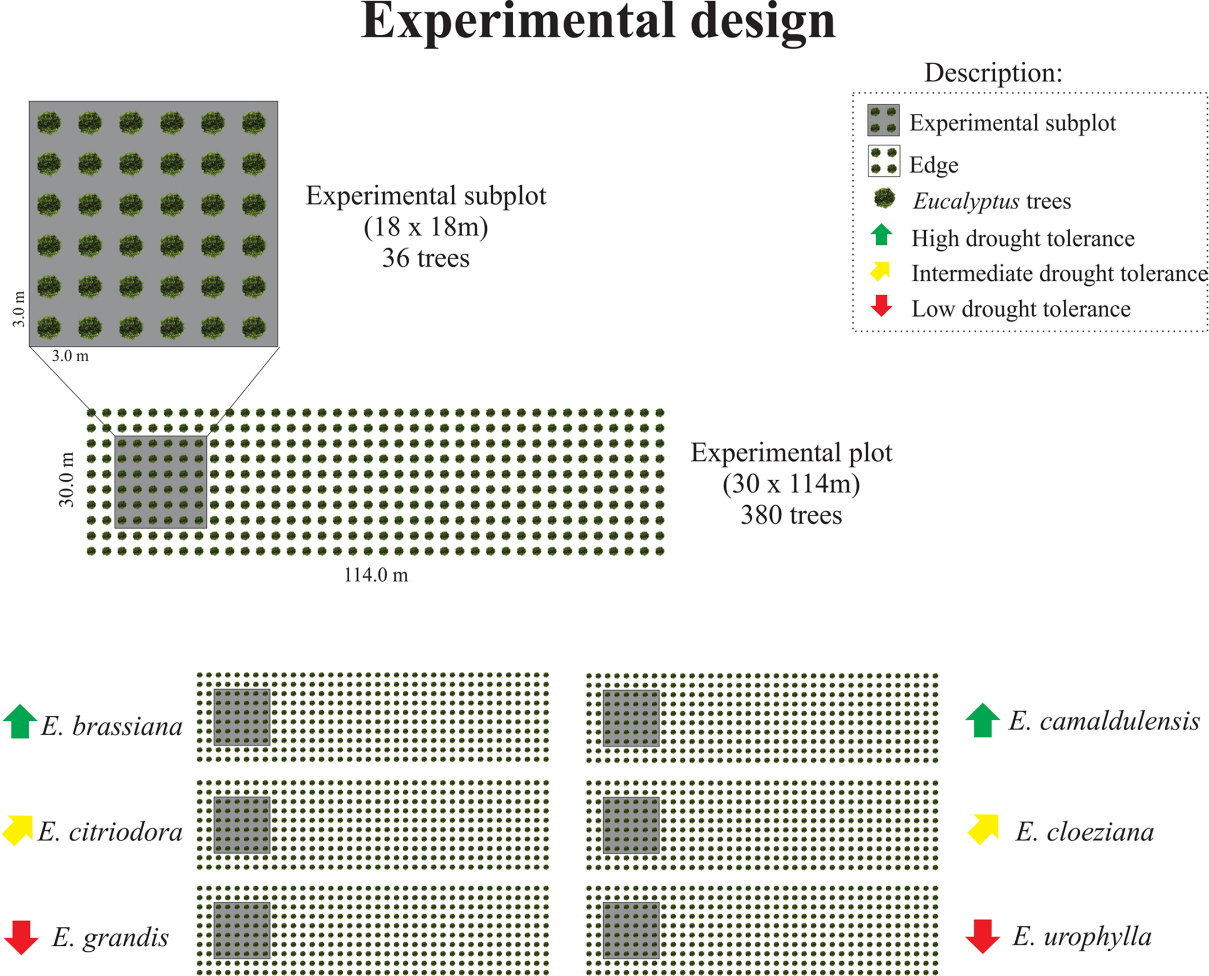
Experimental design showing the plots and subplots in the field considering the different *Eucalyptus* species according to the drought tolerance classification found in Gonçalves et al. (2017).

The experimental design comprised six plots, each planted with one of the following eucalyptus species: *E. brassiana*, *E. camaldulensis*, *E. citriodora*, *E. cloeziana*, *E. grandis*, and *E. urophylla*. These species showed considerable differences in drought tolerance (**Figure 1**; **Table S1**). The area of each plot was 3,420 m² (30 m × 114 m) with 3 x 3m spacing between plants, totaling 380 trees per plot. Some of the trees were used in another study with destructive analyses to evaluate growth parameters. To eliminate the edge effect, sampling was conducted in the central area of each subplot (**Figure 1**).

All plots were fertilized before planting with 2-ton ha^-1^ of limestone, 50 kg ha^-1^ of N, 26.2 kg ha^-1^ of P, and 100 kg ha^-1^ of K. Two after-planting fertilizations were applied in the canopy projection. The first was after four months (July 2016) when 20 kg ha^-1^ of N and 41.6 kg ha^-1^ of K were applied. In the second, 30 kg ha^-1^ of N, 66.5 kg ha^-1^ of K, and 5 kg ha^-1^ of boron were applied 10 months after planting (February 2017).

### 2.2 Soil and Root Sampling

We collected soil and root samples from a depth of 0–10 cm, from the area surrounding five representative trees in each experimental subplot, using the Voronoi polygon approach, according to Pereira et al. (2018). We chose two random points around each tree: one for soil and the other for root sampling. Larger roots were discarded, keeping only the finest and closest roots to the tree base. Samples were obtained in October 2017 and May 2018, when plants were 1.7 and 2.2 years old, respectively. Theoretically, the first period was rainy and the second was dry, although both years were unexpectedly rainy (**Figures S1**, **S2**). At the end of sampling, we obtained 60 soil samples and 60 root samples. Soil samples were sieved (2 mm mesh) and stored at 4°C for chemical and microbiological analyses and at –80°C for molecular analyses. We rinsed the roots with tap water and stored them in tubes containing 70% ethanol at room temperature until staining for arbuscular mycorrhizal fungal colonization analyses.

### 2.3 Soil Physical and Chemical Analyses

Soil pH was measured in 0.01M CaCl_2_ with a 1:2.5 soil-to-water ratio (Minasny et al., 2011). Organic carbon was determined according to Walkley and Black (1934) and macronutrients (nitrogen [ammonium and nitrate], phosphorus, potassium, calcium, magnesium, and sulfur) and micronutrients (copper, iron, manganese, zinc, and boron) were determined according to Raij et al. (2001).

### 2.4 Soil Microbiological Analyses

#### 2.4.1 Microbial Biomass Carbon, Basal Respiration, and Enzyme Activity

Microbial biomass carbon (MBC) was estimated using the fumigation-extraction technique (Vance et al., 1987). Soil basal respiration (BR) was determined by quantifying the CO_2_–C emitted throughout a 25-day incubation at 25°C, according to the method described by Alef and Nannipieri (1995). Dehydrogenase (EC 1.1.1) and acid phosphatase (EC 3.1.3.2) activities were determined as described by Tabatabai and Bremner (1969).

#### 2.4.2 Arbuscular Mycorrhizal Fungi Spores, Root Colonization, and Glomalin-Related Soil Protein

Various parameters were used to assess the presence and activity of AMF. AMF spores were extracted using wet sieving and decanting of 50 g of soil (Gerdemann and Nicolson, 1963).

The roots used to analyze AMF colonization were rinsed with tap water and cut into segments of approximately 1 c. Approximately 0.5 g of these segments was transferred into a 50 mL Falcon^®^ tube, and the roots were covered with a 10% potassium hydroxide (KOH) solution. The tubes were kept in a water bath at 90°C for one hour. Additional bleaching was performed by addition of 10% H_2_O_2_ (alkaline hydrogen peroxide) for 3 min at room temperature. The segments were then colored with blue ink (Parker–Quink) (Vierheilig et al., 1998) for 15 s in a 90°C water bath and kept in tubes containing a lactoglycerol solution (1:1:1 lactic acid, glycerol, and water). Root segments, 1 cm in length, were spread in a petri dish with a 0.5 cm² grid affixed to the base. The total number of root intersections between lines (R1) and the total number of root intersections colonized by mycorrhizal structures (R2) were counted. The ratio of R2/R1 was calculated as the percentage of AMF-colonized roots.

Glomalin-related soil protein (GRSP) is a marker of AM fungal activity. We quantified easily extractable glomalin-related soil protein (EE-GRSP) using the method described by Wright et al. (1996) with some modifications. Air-dried and sieved soil samples (2 mm mesh) were weighed (0.25 g) into 5-mL Eppendorf tubes containing 2 mL of 20 mM sodium citrate pH 7.0, and the resulting suspensions were autoclaved at 121°C for 30 min. After cooling, the suspensions were centrifuged for 10 minutes at 15,000 × *g*. An aliquot of the supernatant was transferred to another tube and centrifuged again for 5 min at 15,000 × *g*. The supernatant was diluted as required (1:2, 1:3, or 1:10) to obtain the absorbance of the sample blank at 465 nm of approximately 0.1, as recommended by Jorge-Araújo et al. (2015), and quantified using the Bradford colorimetric assay, calibrated with solutions of bovine serum albumin (BSA), with concentrations of 0–200 mg L^-1^. All extractions were performed in triplicate, and each extract was assayed in triplicate. The sample solution (20 µL) and Bradford dye reagent (250 µL) were pipetted into the wells. Sample blanks were prepared containing 20 µL of sample extract with 250 µL of 0.1 M HCl (to obtain the same pH as in the reaction mixture). The plate was covered and shaken in a thermomixer for 1 min and then allowed to rest for 10 min. Absorbance was measured at 595 nm, and the protein concentration (equivalent to BSA) was determined after blank sample color correction (Jorge-Araújo et al., 2015).

### 2.5 Soil Molecular Analyses

#### 2.5.1 DNA Extraction

DNA was extracted from 400 mg of each soil sample using the PowerSoil^®^ DNA Isolation kit (MoBio, Carlsbad, CA, USA) following the manufacturer’s instructions. The quality of DNA was checked by agarose gel electrophoresis prior to performing quantitative PCR, and DNA quantity was measured using a Qubit fluorimeter (Invitrogen, Carlsbad, CA).

#### 2.5.2 Quantitative PCR Analysis (qPCR) of the Internal Transcribed Spacer (ITS) Region

ITS region abundance was determined with a real-time PCR system platform (StepOne™) using SYBR^®^ Green PCR Master Mix (Applied Biosystems ^®^), as described by Pereira et al. (2018). The primers used were ITS1F (5’ – CTTGGTCATTTAGAGGAAGTAA – 3’) (Gardes and Bruns, 1993) and 5.8S (5’ – CGCTGCGTTCTTCATCG – 3’) (Gardes and Bruns, 1993). The final volume reaction of 25 µL contained 0.2 µM of both primers, 12.5 µL of SYBR^®^ Green PCR Master Mix (Applied BiosSystems^®^), 1 µL DNA template, and 11.1 µL DNA free water. The thermal cycling conditions were 94°C for 15  min and 40 amplification cycles of 94°C for 60 s, 53°C for 30 s, and 72°C for 60 s. The ITS region copy numbers were calculated by interpolating the value of cycles obtained per sample (CT = cycle threshold) in the linear regression generated by the standard dilution series from 10 to 10^6^. Only reactions with R^2^ values ≥ 0.98 were taken into consideration. The efficiency (E) of the qPCR was 102%, calculated using the equation E = [10 (−1/slope)^-1^.

#### 2.5.3 Terminal Restriction Fragment Length Polymorphism (T-RFLP) of the ITS Region

The fungal community structure was obtained by T-RFLP using the amplified soil DNA with the primers ITS1F-FAM (5′– CTTGGTCATTTAGAGGAAGTAA – 3′) (Gardes and Bruns, 1993) and ITS4 (5′ – TCCTCCGCTTATTGATATC – 3′) (White et al., 1990) in triplicate. The final reaction volume (50 µL) contained 6 mM MgCl_2_, 4 mM dNTP (Invitrogen Corporation, USA), 0.5 U μL^-1^ of Taq polymerase (Sinapse, São Paulo, Brazil), buffer for Taq polymerase 1X, 0.20 mmol μl of both primers, 1.5 g L^-1^ bovine serum albumin (BSA), 2 μL of DNA template, and DNA free water to complete the reaction volume. The thermal cycling conditions were as follows: 94°C for 60 s; 13 amplification cycles of 94°C for 35 s, 55°C for 55 s, and 72°C for 45 s; 13 amplification cycles of 94°C for 35 s, 55°C for 2 min, and 72°C for 45 s; 9 amplification cycles of 94°C for 35 s, 55°C for 3 min, and 72°C for 45 s; and a final extension at 72°C for 10 min. After PCR amplification, the samples were purified using 75% isopropanol and quantified on 2% agarose gel. To make cleavages with the respective endonucleases, we used the PCR product with 5U of the enzyme HaeIII (Thermo Scientific, Massachusetts, USA), following the manufacturer’s instructions. The products were precipitated with sodium acetate 3 M and EDTA 125 mM, resuspended in Hidi formamide with the marker LIZ 600 (Applied Biosystems), and each sample was read on an automatic sequencer ABI Prism 3500As (Applied Biosystems). A threshold of 50 units of fluorescence was adopted to remove the “background” of the samples, and the results were transformed into a relative abundance matrix of the peak areas.

### 2.6 Statistical Analyses

Data from both samples were combined and analyzed together, as they were not significantly different among the sampling periods (**Figures S1**, **S2**). We used the Kruskal–Wallis method as a non-parametric test to investigate the effects of *Eucalyptus* species on soil chemical and microbiological attributes, including fungal richness and diversity. The significance level set was 0.1 (10%), considering that the experiment was carried out under field conditions (Fageria, 2007; Tavares et al., 2016). We adopted a non-parametric approach because the data residues did not meet the assumptions for ANOVA (homogeneity and normality) using the *Agricolae* package (Mendiburu, 2020).

We evaluated the changes in the soil fungal community structure based on the Bray-Curtis distances of the relative abundance of the peak area matrix obtained from the terminal restriction fragment length polymorphism (T-RFLP). Shannon–Weaver and richness indices of the terminal restriction fragments (T-RFs) were calculated according to Zhang et al. (2008). To determine the differences in the soil fungal community structure between the *Eucalyptus* species, we performed a PERMANOVA analysis (Adonis function in R, Permutations = 999), considering the relative abundance of the peak area matrix transformed into log (x+1) (Ramette, 2007). We carried out a global redundancy analysis (RDA) coupled with a *forward selection* function to verify the correlations between AMF groups and soil chemical properties. Diversity, richness, and PERMANOVA were performed using the *vegan* package (Oksanen et al., 2008).

A structural equation model (SEM) was adopted to explore how the soil microbiological attributes influenced the soil fungal community structure for each *Eucalyptus* species. First, we used non-metric multidimensional scaling (NMDS) to obtain the first axis scores, which were used as indicators of fungal community structure (composite variable) (Song et al., 2013; Ling et al., 2017; Vries et al., 2018; Yang et al., 2020). We used a minimum set of parameters to assess the model fit, including root mean square error of approximation (RMSEA), comparative fit index (CFI), Tucker-Lewis index (TLI), and standardized root mean square residual (SRMR), with the benchmark values according to Fan et al. (2016). The modeling process was performed using *lavaan* (Rosseel, 2012) and *semPlot* (Epskamp, 2015) packages. Additionally, we performed Spearman’s rank correlation between soil chemical attributes and soil fungal community structure for each *Eucalyptus* species using the package *corrplot* (Wei, 2017). All statistical analyses were performed using the R software version 3.3.2 (R Core Team, 2016).

## 3 Results

### 3.1 Soil Chemical Analyses

There was no difference in either the total rain accumulated six months before each sampling period (**Figure S1**) or in the soil moisture between the sampling periods (October 2017 and May 2018) (**Figure S2**). For this reason, we could not consider the effect of moisture, as originally intended. We attempted to evaluate most of the possible interactions between *Eucalyptus* species, AMF, and soil chemical and microbiological characteristics. Although our results do not always help explain many of the underlying mechanisms, we believe that they may contain certain hints, which can be helpful in future studies related to our topic.

The contents of most of the macro- and micronutrients showed significant differences between *Eucalyptus* species, except for pH, Al, Mg, Ca, and Fe contents in the soil (**Table 1**). We also observed differences in the potential acidity (H + Al) and cation exchange capacity (CEC), both of which were higher in the presence of *E. urophylla* (respectively, 57.3 and 73.3 mmol_c_ kg^-1^). Soil organic carbon was higher in the presence of *E. camaldulensis* (21.2 g kg^-1^), whereas the lowest levels were detected in *E. urophylla* (20.4* g* kg^-1^) and *E. grandis* (16.0 g kg^-1^). Soil ammonium (NH^4+^) content was approximately 2-fold higher in *E. grandis* than in *E. camaldulensis* and *E. citriodora*. In the soil with *E. camaldulensis* we observed the lowest nitrate content (9.69 mg kg^-1^) among all *Eucalyptus* species (mean of 16.9 mg kg^-1^) (**Table 1**). However, in many cases, there were no significant differences between species.

**Table 1:** Soil chemical attributes in *Eucalyptus* species.

Eucalyptus species	pH	Al	OC	NH_4_^+^	NO_3_^-^
	CaCl_2_	mmol_c_ kg^-1^	g kg^-1^	—————mg kg^-1^—————	
*E. brassiana*	4.38 ± 0.42 a	4.42 ± 2.81 a	18.97 ± 3.96 ab	23.07 ± 7.50 ab	15.07 ± 5.98 a
*E. camaldulensis*	4.24 ± 0.39 a	5.64 ± 4.09 a	21.19 ± 3.25 a	18.75 ± 12.65b	9.69 ± 3.94 b
*E. citriodora*	4.29 ± 0.50 a	4.96 ± 3.55 a	19.55 ± 4.33 ab	21.63 ± 11.71b	15.38 ± 7.02 a
*E. cloeziana*	4.33 ± 0.51 a	5.00 ± 4.51 a	20.00 ± 7.65 ab	28.75 ± 20.40ab	18.38 ± 6.88 a
*E. grandis*	4.25 ± 0.38 a	4.44 ± 2.93 a	16.02 ± 3.20 b	41.32 ± 24.35a	18.94 ± 8.01 a
*E. urophylla*	4.11 ± 0.43 a	6.38 ± 3.86 a	20.42 ± 10.70b	25.44 ± 13.41ab	16.63 ± 7.67 a
	P	K	Ca	Mg	S
	mg kg^-1^	————————mmol_c_ kg^-1^————————–			mg kg^-1^
*E. brassiana*	3.88 ± 2.05 a	0.51 ± 0.19 a	9.11 ± 5.52 a	5.25 ± 3.35 a	4.01 ± 1.97ab
*E. camaldulensis*	3.01 ± 1.32 ab	0.45 ± 0.09 a	8.75 ± 4.16 a	5.38 ± 2.79 a	4.51 ± 2.28a
*E. citriodora*	2.67 ± 1.12 ab	0.51 ± 0.14 a	10.60 ± 10.94a	6.26 ± 6.56 a	3.69 ± 1.34ab
*E. cloeziana*	6.32 ± 7.24 ab	0.32 ± 0.15 bc	7.76 ± 3.68 a	4.63 ± 2.00 a	2.93 ± 0.71b
*E. grandis*	3.41 ± 3.98 b	0.27 ± 0.08 c	8.37 ± 5.39 a	6.14 ± 4.38 a	3.95 ± 1.76ab
*E. urophylla*	4.22 ± 4.71 ab	0.66 ± 0.77 ab	9.29 ± 10.02a	6.10 ± 6.40 a	3.81 ± 1.12b
	SB	H+Al	CEC	V	m
	————————mmol_c_ kg^-1^————————			——————– % —————–	
*E. brassiana*	14.87 ± 8.72 a	47.30 ± 16.01 ab	62.17 ± 13.07 ab	24.72 ± 15.30 a	27.29 ± 19.53 a
*E. camaldulensis*	14.58 ± 6.68 a	48.70 ± 18.17 ab	63.28 ± 17.11 ab	24.31 ± 12.26 a	29.46 ± 20.66 a
*E. citriodora*	17.37 ± 17.36a	46.00 ± 20.05 ab	63.37 ± 19.20 b	26.42 ± 19.18 a	29.55 ± 20.86 a
*E. cloeziana*	12.72 ± 5.51 a	45.80 ± 25.19 b	58.52 ± 23.63 b	25.13 ± 13.86 a	28.38 ± 24.14 a
*E. grandis*	14.77 ± 9.61 a	40.10 ± 13.23 b	54.87 ± 11.79 b	26.81 ± 15.50 a	28.59 ± 21.17 a
*E. urophylla*	16.05 ± 16.79a	57.30 ± 19.71 a	73.35 ± 20.74 a	21.21 ± 20.50 a	42.21 ± 28.97 a
	Cu	Fe	Mn	Zn	B
	—————————————————–mg kg^-1^—————————————————–				
*E. brassiana*	0.82 ± 0.18ab	194.1 ± 100.1 a	2.07 ± 0.93 b	0.08 ± 0.07 a	0.22 ± 0.10 a
*E. camaldulensis*	0.61 ± 0.34b	175.5 ± 128.7 a	2.56 ± 1.66 ab	0.06 ± 0.07 ab	0.19 ± 0.05 ab
*E. citriodora*	0.78 ± 0.12ab	206.8 ± 133.6 a	2.48 ± 1.31 ab	0.02 ± 0.02 b	0.20 ± 0.05 a
*E. cloeziana*	0.69 ± 0.14b	205.9 ± 178.0 a	3.26 ± 1.35 a	0.10 ± 0.12 a	0.16 ± 0.03 b
*E. grandis*	0.70 ± 0.11b	182.2 ± 113.8 a	2.69 ± 1.02 ab	0.05 ± 0.06 ab	0.16 ± 0.02 b
*E. urophylla*	0.85 ± 0.19a	262.8 ± 166.3 a	3.32 ± 3.37 ab	0.08 ± 0.17 b	0.19 ± 0.04 a

Means followed by standard deviations. Lowercase letters compare the differences among Eucalyptus species using the Kruskal–Wallis test by rank (p ≤ 0.1). pH, active acidity; Al, aluminum; OC, organic carbon; NH_4_^+^, ammonium; NO_3_^–^, nitrate; P, phosphorus; K, potassium; Ca, calcium; Mg, magnesium; S, sulfur; SB, sum of bases (Ca, Mg, and K); H+Al, potential acidity; CEC, cation exchange capacity; V, base saturation; m, saturation by aluminum for effective CEC; Cu, copper; Fe, iron; Mn, manganese; Zn, zinc; B, boron. mmol_c_ kg^-1^: millimoles of charge per kilogram of soil according to SI unit (International Standard of Units).

Phosphorus (P) and potassium (K) contents in soil were higher in the presence of *E. brassiana* (3.9 mg kg^-1^ and 0.5 mmol_c_ kg^-1^, respectively) and lower in the presence of *E. grandis* (3.4 mg kg^-1^ and 0.3 mmol_c_ kg^-1^ for P and K, respectively) (**Table 1**). Soil K content did not differ among *E. brassiana*, *E. camaldulensis*, and *E. citriodora*. Sulfur content in soil was high in the presence of *E. camaldulensis* (4.5 mg kg^-1^) differing from both *E. cloeziana* (2.9 mg kg^-1^) and *E. urophylla* (3.8 mg kg^-1^). Copper and boron content in the soil was higher in the presence of *E. urophylla* than in *E. grandis* and *E. cloeziana*, although there was no difference in boron content among *E. urophylla*, *E. brassiana*, and *E. citriodora*. Manganese content in soil was higher in the presence of *E. cloeziana* (3.3 mg kg^-1^), showing a lower content in the presence of *E. brassiana* (2.1 mg kg^-1^), while a higher zinc content (approximately 0.1 mg kg^-1^) was observed in soil with these same *Eucalyptus* species (*E. cloeziana* and *E. brassiana*).

### 3.2 Soil Microbiological Analyses

Microbial biomass carbon (MBC) and basal respiration (BR) did not differ in soil with different *Eucalyptus* species (**Table 2**). In general, MBC ranged between 125.8 (for *E. citriodora*) and 175.26 mg C g soil^-1^ (for *E. urophylla*). The BR values ranged from 18.0 (for *E. brassiana*) to 24.9 mg C–CO_2_ kg soil^-1^ day^-1^ (for *E. urophylla*).

**Table 2:** Soil microbiological attributes in *Eucalyptus* species.

Eucalyptus species	AMF Spores	AMF Col	Glom	Phos Actv
	50 g soil^-1^	%	g kg soil^-1^	µg PNF g^-1^ soil h^-1^
*E. brassiana*	44.60 ± 32.31 a	12.10 ± 12.48 c	1.16 ± 0.46 a	469.88 ± 144.21 b
*E. camaldulensis*	17.20 ± 20.05 b	23.40 ± 14.92 ab	1.19 ± 0.33 a	587.48 ± 148.54 ab
*E. citriodora*	23.90 ± 28.43 ab	23.50 ± 19.06 abc	1.09 ± 0.23 a	553.53 ± 140.90 ab
*E. cloeziana*	31.50 ± 41.23 ab	10.90 ± 7.28 bc	1.44 ± 0.66 a	646.67 ± 267.23 a
*E. grandis*	13.60 ± 13.81 b	25.10 ± 24.37 abc	1.10 ± 0.31 a	501.90 ± 178.74 ab
*E. urophylla*	39.20 ± 49.52 ab	30.90 ± 18.82 a	1.20 ± 0.62 a	574.28 ± 245.97 ab
	Dehy Actv	MBC	Basal Resp	qPCR ITS
	µg TTF g^-1^ soil day^-1^	mg C g soil^-1^	mg C-CO_2_ kg soil^-1^ day^-1^	Copy number g soil^-1^
*E. brassiana*	2.17 ± 0.22 a	138.78 ± 64.02 a	18.03 ± 16.28 a	7.13E+07 ± 3.92E+07 ab
*E. camaldulensis*	2.15 ± 0.51ab	166.77 ± 79.42 a	22.34 ± 18.81 a	1.49E+08 ± 1.31E+08 a
*E. citriodora*	1.99 ± 0.05 b	125.80 ± 67.03 a	18.52 ± 13.62 a	8.97E+07 ± 5.23E+07 ab
*E. cloeziana*	2.04 ± 0.10 ab	150.10 ± 51.68 a	22.09 ± 22.15 a	8.49E+07 ± 5.48E+07 ab
*E. grandis*	2.03 ± 0.17 b	148.56 ± 75.31 a	19.24 ± 13.14 a	1.02E+08 ± 8.38E+07 ab
*E. urophylla*	2.08 ± 0.15 ab	175.26 ± 84.45 a	24.87 ± 16.43 a	6.26E+07 ± 3.71E+07 b

Means followed by standard deviations. Lowercase letters compare the differences among Eucalyptus species using the Kruskal–Wallis test by rank (p ≤ 0.1). AMF Spores, arbuscular mycorrhizal fungi (AMF) spore numbers; AMF Col, root colonization by AMF; Glom, easily extractable glomalin-related soil protein; Phos Actv, acid phosphatase activity; Dehy Actv, dehydrogenase activity; MBC, microbial biomass carbon; Basal Resp, basal respiration; qPCR ITS, ITS copy number in soil.

Differences in soil enzyme also observed. Acid phosphatase activity was 1.4–fold higher in the presence of *E. cloeziana* (646 µg PNF g^-1^ soil) than in *E. brassiana* (469.88 µg PNF g^-1^ soil). On the other hand, in the presence of *E. brassiana*, we observed higher dehydrogenase activity (2.2 µg TTF g^-1^ soil day^-1^), when compared with *E. citriodora* and *E. grandis* (2.0 µg TTF g^-1^ soil day^-1^).

Higher AMF spore numbers (approximately 45 spores) were present in soil planted with *E. brassiana*, whereas in soil with *E. camaldulensis* and *E. grandis* we found the lowest values (approximately 17 and 14, respectively). Despite the higher AMF spore numbers in soil, *E. brassiana* had the lowest AMF root colonization (10.9%) among the six *Eucalyptus* species. Meanwhile, the highest AMF colonization was observed in *E. urophylla* (30.9%). There was no difference in easy extractable glomalin-related soil protein, ranging from 1.1 g kg soil^-1^ (for *E. citriodora*) to 1.4 g kg soil^-1^ (for *E. cloeziana*). The greatest abundance of the ITS regions in the soil was in the presence of *E. camaldulensis* (1.5 10^8^ ITS copy numbers g soil^-1^), whereas the lowest values were detected in the presence of *E. urophylla* (6.3 10^7^ ITS copy numbers g soil^-1^) (**Table 2**).

### 3.3 Associations Among the Soil Fungal Community Structure and Soil Microbiological and Chemical Attributes

**Figure 2:**
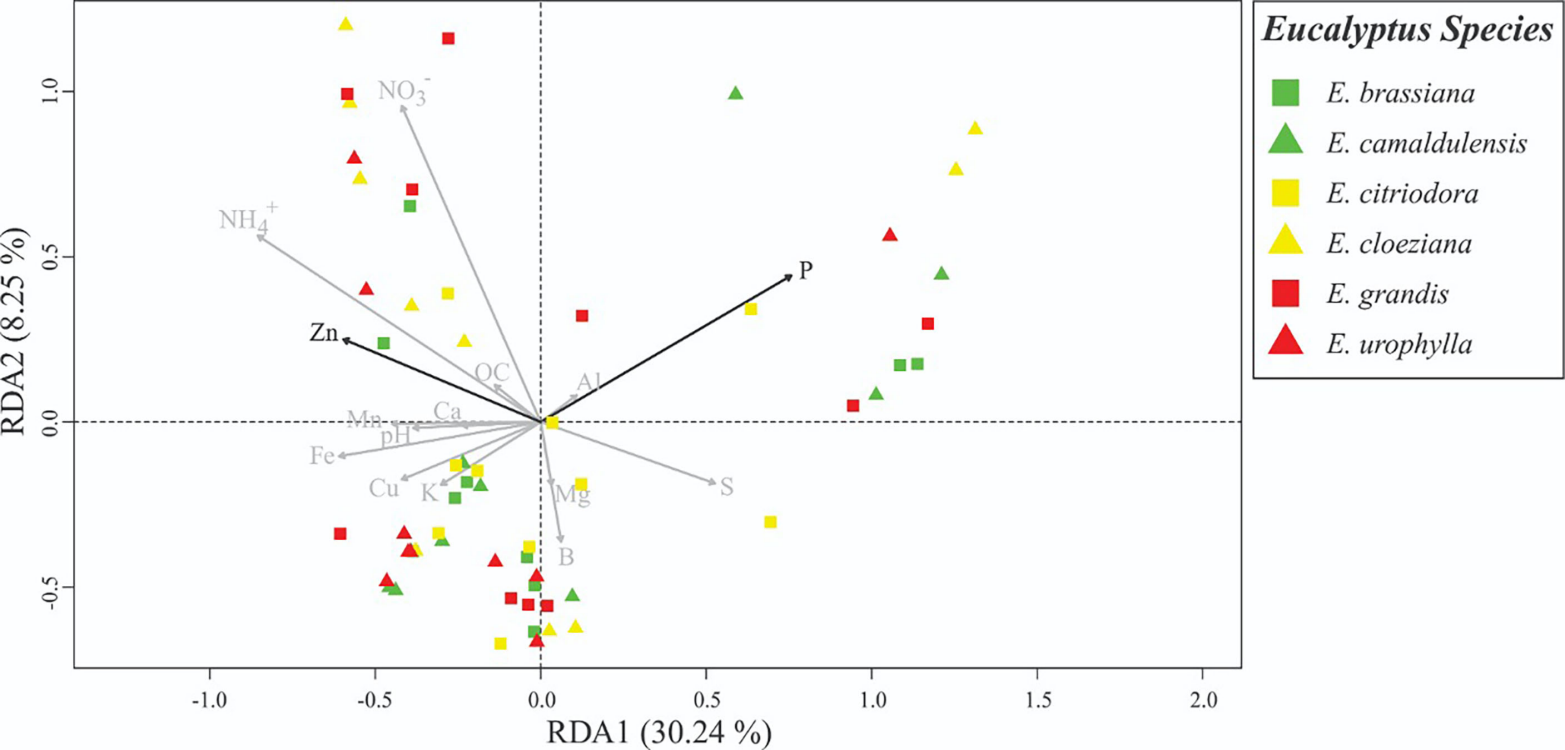
Redundancy analysis (RDA) showing the correlations between AMF-groups and soil chemical properties. Black arrows indicate the significant attributes detected through forward selection.

**Figure 3:**
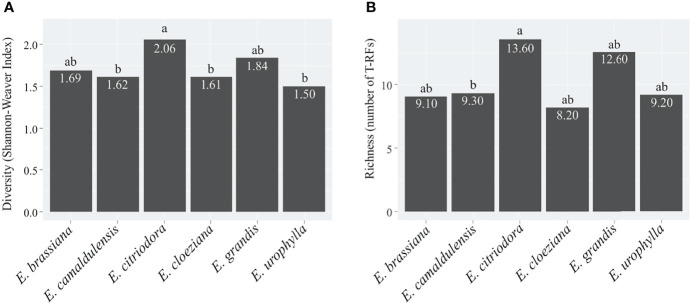
Diversity **(A)** and Richness **(B)** of the soil-fungal community structure in plots with different *Eucalyptus* species based on terminal restriction fragments (T-RFs). Indexes considering the matrix of the peak area obtained by terminal restriction fragment length polymorphism of the soil fungal community structure in different *Eucalyptus* species plantations. Numbers within the bars denote the mean value of each *Eucalyptus* species and lowercase letters represent the comparison of different *Eucalyptus* species by the Kruskal–Wallis test by ranks (p ≤ 0.1).

To detect changes in the soil fungal community structure, we applied a fingerprinting approach based on the terminal restriction fragment length polymorphism (T-RFLP) of the ITS region. We applied NMDS to verify differences in the mycorrhizal community structure between different treatments with *Eucalyptus* species, as well as to obtain the first axis scores, which were used as indicators of the fungal community structure (**Figure S3**). According to redundancy analysis (RDA), there was a significant difference (F = 1.6413, p = 0.006) in the AMF structure. In this case, the Zn (p = 0.017) and P (p = 0.013) contents were the most important drivers of community changes (**Table S2**). The most significant differences in soil fungal community structure were observed for *E. citriodora*, *E. cloeziana*, and *E. urophylla* (**Figure 2**). The greatest diversity (based on terminal restriction fragments) occurred in the presence of *E. citriodora* and the lowest diversity occurred in soil with *E. cloeziana* and *E. urophylla* (**Figure 3**).

To test for relationships between changes in chemical and microbiological attributes of soil and soil fungal community structure, we adopted two approaches with different sensitivities. First, we applied structural equation models to assess the effect of soil microbiological attributes on soil fungal community structure. Second, we used Spearman’s rank correlation to find different correlation dynamics between soil chemical attributes and soil fungal community structure.

In summary, the soil fungal community structure differed only between *E. citriodora* and *E. cloeziana* (R² = 0.16, p < 0.01) and between *E. citriodora* and *E. urophylla* (R² = 0.18, p < 0.01) (**Table 3**). We also observed that fungal T-RF diversity (**Figure 3A**) and richness (**Figure 3B**) were higher in the presence of *E. citriodora* while the lowest T-RF diversity values were present in *E. camaldulensis*, *E. cloeziana*, and *E. urophylla* (**Figure 3A**). The lowest fungal T-RF richness was observed in *E. camaldulensis* (**Figure 3B**).

**Table 3:** R² value obtained by Adonis test among *Eucalyptus* species using the dissimilarity matrix (Bray-Curtis distance) of the soil fungal community structure obtained by terminal restriction fragment length polymorphism.

	*E. brassiana*	*E. camaldulensis*	*E. citriodora*	*E. cloeziana*	*E. grandis*	*E. urophylla*
*E. brassiana*	1.00					
*E. camaldulensis*	0.03	1.00				
*E. citriodora*	0.09	0.03	1.00			
*E. cloeziana*	0.04	0.09	0.16**	1.00		
*E. grandis*	0.02	0.00	0.03	0.08	1.00	
*E. urophylla*	0.03	0.10	0.18**	0.03	0.09	1.00

**(p < 0.01).

Bold values indicate the only significant pairwise comparisons obtained by the Adonis test.

**Figure 4:**
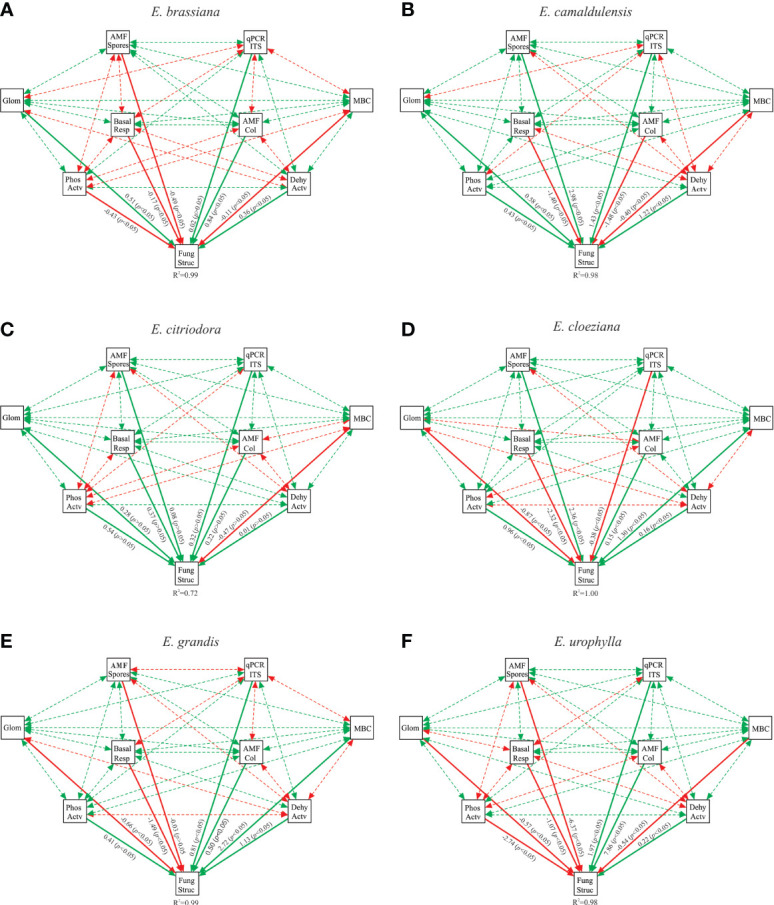
Structural equation model of correlations between soil microbiological attributes and the soil fungal structure community for Eucalyptus brassiana **(A)**, *E. camaldulensis* **(B)**, *E. citriodora* **(C)**, *E. cloeziana* **(D)**, *E. grandis* **(E)**, and *E. urophylla* **(F)**. Values associated with solid arrows represent significant standardized path coefficients. Solid or dashed arrows indicate direct or indirect correlation, respectively. Red arrows indicate negative correlation while green arrows indicate positive correlation. The R2 values associated with the fungal structure indicate the proportion of variation explained by correlations with other variables. AMF Spores, arbuscular mycorrhizal fungi (AMF) spore numbers; AMF Col, root colonization by AMF; Glom, easily extractable glomalin-related soil protein; Phos Actv, acid phosphatase activity; Dehy Actv, dehydrogenase activity; MBC, microbial biomass carbon; Basal Resp, basal respiration and qPCR ITS, ITS copy number in soil.

We built a structural equation model (SEM) to assess the direct and indirect effects of the microbiological attributes of the soil fungal community structure on each *Eucalyptus* species. According to a set of parameters assessed to fit the model (RMSEA, CFI, TLI, and SRMR), our modeling was classified as excellent considering the benchmark values (**Table S3**). In general, we found that soil microbiological attributes affected the soil fungal community structure differently for each *Eucalyptus* species (**Figure 4**).

Considering the highest standardized path coefficients, we found that in *E. brassiana*, AMF colonization and glomalin correlated positively with the fungal community structure, whereas the AMF spore number and phosphatase activity were negatively correlated (**Figure 4A**). In addition, the highest number of indirect negative interactions between soil microbiological attributes was observed in *E. brassiana* (**Table S4**). *E. camaldulensis* had one of the highest standardized path coefficients between fungal community structure and AMF spore number, as well as the abundance of the ITS region. In general, there were a greater number of positive correlations in *E. camaldulensis* (**Figure 4B** and **Table S4**). It is striking that the soil fungal community structure was positively correlated with all soil microbiological attributes, except for microbial biomass carbon, in the presence of *E. citriodora*. Phosphatase activity and soil basal respiration showed the highest standardized path coefficients (**Figure 4C**). In the presence of *E. cloeziana*, the soil fungal community structure was positively correlated with AMF spore number, microbial biomass carbon, and phosphatase activity; and negatively correlated with soil basal respiration and EE-GRSP (**Figure 4D**). At the same time, the presence of *E. cloeziana* provided the greatest positive indirect correlations among the soil microbiological attributes (**Table S4**). We found that soil basal respiration was the major microbiological attribute, which was negatively correlated with the soil fungal community structure, whereas the microbial biomass carbon and dehydrogenase activity correlated positively with *E. grandis* (**Figure 4E**). Equally, the highest standardized path coefficient appeared among the soil fungal community structure and soil microbiological attributes of *E. urophylla*. In general, there were more negative and positive correlations with *E. urophylla*.

**Figure 5:**
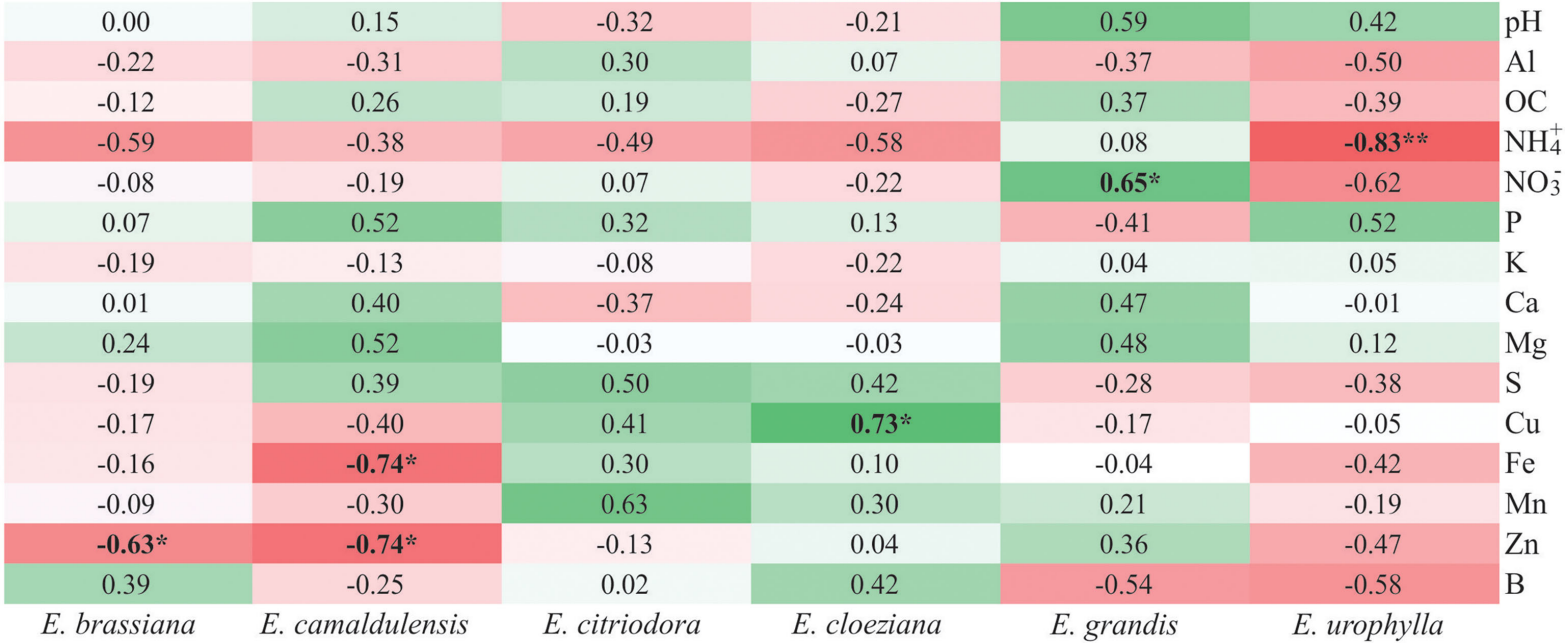
Heatmap considering Spearman’s rank correlation coefficient between soil fungal community structure and soil chemical attributes in different *Eucalyptus* species. Red color indicates a negative correlation while the green color indicates a positive correlation. The intensity of the colors indicates the strength of the correlations. *p < 0.05, **p < 0.01.

We associated soil chemical attributes with the soil fungal community structure using Spearman’s rank correlation (**Figure 5**) and found different trends among the *Eucalyptus* species. In general, we observed that in the presence of *E. urophylla*, there were only negative correlations with soil nutrient content, and the only significant correlation occurred between soil ammonium content and soil fungal community structure (R = –0.83, p < 0.01).

In the presence of *E. grandis* there was a positive correlation between soil nitrate content and soil fungal community structure (R = 0.65, p < 0.05). The greatest number of positive correlations between the soil chemical attributes and soil fungal community structure was detected in the presence of *E. citriodora* followed by *E. cloeziana*. In *E. cloeziana*, we also detected that all correlations between the soil fungal community structure and soil micronutrients were positive, with copper having the strongest correlation (R = 0.73, p < 0.05). Negative correlations were observed between soil zinc content and soil fungal community structure in the presence of *E. brassiana* (R = –0.63, p < 0.05) and *E. camaldulensis* (R = –0.74, p < 0.05). Additionally, the latter showed a negative correlation with soil iron content (R = –0.74, p < 0.05) (**Figure 5**).

## 4 Discussion

Given the paucity of information on the effect of different *Eucalyptus* species on the fungal community structure and soil chemical and microbiological attributes, our study showed evidence of several significant correlations between them. This is especially true in the context of the genotype effect, since different drought tolerance levels seem to have a certain correlation with some interactions of each tree species, as described by Gonçalves et al. (2017). Although we were unable to explain the mechanisms for each negative or positive interaction and the dynamics of soil chemical and microbiological attributes in this exploratory study, it may be a preliminary observation whose follow-up may elucidate some remaining questions. This report presents different ways in which symbioses could interact between AMF and *Eucalyptus*. It is crucial to understand the soil fungal community, especially in the context of a bio-based circular bioeconomy, in which the fungus appears as a biotechnological tool that is critical for the functioning and health of forests (Meyer et al., 2020; Steidinger et al., 2020).

### 4.1 Soil Chemical Analyses

Differences in soil nutrient contents (macro- and micronutrients) reflect the influence of *Eucalyptus* species on soil nutrient dynamics, which may be related to differences in the quantity and quality of litter deposition, in addition to the root exudate composition (Brunner et al., 2015). In our study, higher organic carbon and lower nitrogen rates were found in soils with *E. camaldulensis*, resulting in a high soil C/N ratio, which should lead to temporary nutrient immobilization in soil microbial biomass (Goya et al., 2008). The opposite was found in soil with *E. grandis*, which presented lower organic carbon and higher nitrogen content, leading to a low soil C/N ratio. The different C/N ratios correlate with total litter deposition, which increases the organic matter in the soil. These *Eucalyptus* species have different drought tolerance levels; *E. camaldulensis* is considered a species with high drought tolerance (and a high C/N ratio), whereas *E. grandis* is a low drought-tolerant species (with a low C/N ratio) (Gonçalves et al., 2017). Despite the molecular and anatomical adaptations of each species, soil organic carbon has a significant influence on drought tolerance due to an improvement in several soil properties, such as greater soil aggregation stability and greater soil porosity with better rainwater infiltration (Bot and Benites, 2005; Brunner et al., 2015).

Some authors have suggested different litter depositions for stands of *E. camaldulensis* and *E. grandis*. Silva et al. (2015) reported a total litter deposition in soil for stands of *E. camaldulensis* of 6.3 Mg ha^-1^ year^-1^, while Vargas et al. (2019) found stands of 2.9 Mg ha^-1^ year^-1^ for *E. grandis*, i.e., 2.17-fold less litter deposition in *E. grandis* than in *E. camaldulensis*. Although we did not assess litter quality, different *Eucalyptus* species are known to have different litter compositions, which may influence both microbial activity and nutrient cycling (Nogueira et al., 2006; Yang et al., 2020). In addition, Chabrerie et al. (2003) and Sinha et al. (2009) considered the quality and quantity of rhizodeposition of different species to be a more important factor than climatic conditions for the modulation of the microbial community.

Overall, our study also showed a species effect on soil macro- and micronutrients, as revealed by the significant increase in soil phosphorus and K (especially in *E. brassiana*), soil sulfur (in *E. camaldulensis*), copper and boron (in *E. urophylla*), and manganese (in *E. cloeziana*).

### 4.2 Soil Microbiological Analyses

Soil enzyme activity, arbuscular mycorrhizal fungi (spore numbers and colonization), and ITS region abundance were the most responsive soil microbiological attributes, suggesting a functional effect on the soil fungal community by *Eucalyptus* species. We observed inverse dynamics between soil acid phosphatase and dehydrogenase activity among *Eucalyptus* species. The main producers of soil enzymes are microorganisms, followed by plants (Rao et al., 2014). Phosphatase release is usually enhanced by the limited availability of P in the soil (Raghothama and Karthikeyan, 2005). We found that *E. brassiana* showed the lowest soil acid phosphatase activity and soil AMF root colonization rate related to the highest soil phosphorus content, as previously described (Bini et al., 2018; Pereira et al., 2018). According to Liu et al. (2016), high soil P content reduces root AMF colonization, whereas low P content tends to increase colonization and fungal richness. In addition, *E. brassiana* and *E. camaldulensis* are highly drought-tolerant species; however, the growth rates of *E. brassiana* are generally higher than those of *E. camaldulensis* (Gonçalves et al., 2017; **Table S1**).

We observed that in the presence of *E. brassiana* there was greater dehydrogenase activity, high soil organic carbon content, and high AMF spore numbers. It is well known that dehydrogenase is an enzyme involved in the mineralization of organic matter and the release of nutrients, that is, there is a positive correlation between dehydrogenase and microbial activity (Bini et al., 2018).

*E. urophylla* was the species with the most AMF colonization (30.9%), indicating a very responsive behavior to AMF, as well as a low tolerance to low moisture. Although soil moisture did not vary during the sampling period, a greenhouse assay carried out by the first author with the same *Eucalyptus* species and soil type under three different moisture levels showed greater AMF colonization of *E. urophylla* (unpublished data). Campos obtained similar results with 26% of AMF colonization in *E. urophylla* (Da Campos et al., 2011). Another trait of this species is the presence of putative allelochemicals that can affect soil microbiota. However, Qin and Yu (2019) demonstrated that the allelochemicals of *E. urophylla* do not affect mycorrhizal symbiosis. In contrast, the authors found evidence of a positive correlation between allelopathy and mycorrhizal growth. Despite the greater AMF colonization, the lowest soil fungal community, evaluated by quantitative PCR, was observed for *E. urophylla* and there were negative correlations with the majority of other soil microbe indicators (discussed in the next section). In this survey, we found species-specific influences of *Eucalyptus* on mycorrhizal traits (spore number and root colonization), as observed by other authors (Soumare et al., 2015; Klinsukon et al., 2020).

### 4.3 Correlating Soil Fungal Community Structure With Soil Microbiological and Chemical Attributes

In our interpretation of SEM, we considered only the highest standardized path coefficients; however, according to Domeignoz-Horta et al. (2020), caution is necessary when drawing conclusions from the path signal. In general, microbiological attributes do not exert the same influence on soil fungal community structure in different *Eucalyptus* species. AMF spore number correlates positively with the soil fungal community structure only in the presence of *E. camaldulensis* and *E. cloeziana*, which are considered to be the most tolerant to drought (Gonçalves et al., 2017). In contrast, AMF spore number was negatively correlated with soil fungal community structure only in the presence of *E. brassiana* and *E. urophylla*, which are the most susceptible to drought. Lovelock and Ewel (2005) observed that the relative abundance of AMF spores was affected by the host tree species in tropical ecosystems.

However, fungal communities (including AMF) directly or indirectly affect plant dispersal and competition, in addition to regulating plant coexistence and diversity at a local scale (Tedersoo et al., 2020). In this sense, our research suggests that AMF colonization of all the tested *Eucalyptus* species, except for *E. camaldulensis*, correlates positively with the soil fungal community structure. However, it was evident that AMF sporulation (assessed by AMF spore number) was most strongly associated with high or intermediate drought tolerant *Eucalyptus* while high AMF root colonization intensity was associated with low drought tolerance. In addition, we observed that the main positive chemical driver of the soil fungal community structure in the presence of *E. cloeziana* was the copper content in the soil, whereas it was the soil nitrate content in the presence of *E. grandis*. Although differences were detected in the root colonization percentages and spore numbers between *Eucalyptus* species, we cannot affirm that AMF had any effect on them. Obviously, it is impossible to demonstrate their degree of effectiveness in protecting trees against water scarcity in the soil. The cause of this may be, as already explained, that unfortunately in the crucial sampling year there was no difference detected between the soil moisture in the two soil sampling periods, although theoretically one of them belonged to the rainy period and the other to the dry period.

Considering both soil chemical attributes and microbiological findings, we found that both seemed to play a positive role when interacting with *E. citriodora*. This may indicate that both types of interactions contribute to the activities of the fungal community. On the other hand, in the presence of *E. urophylla*, these attributes may have had a negative effect on the soil fungal community; probably, these attributes were less likely to have driven changes in the fungal community. In addition, we found species-specific correlations between soil fungal community structure and soil chemical attributes. Thus, a shift in the soil fungal community may have a strong influence on the dynamics of soil nutrients.

## 5 Conclusions

Soil organic carbon, phosphorus, potassium, and some micronutrients (zinc, copper, and iron) appear to correlate with changes in the soil fungal community structure in the presence of *E brassiana*, *E. camaldulensis*, *E. citriodora*, and *E. cloeziana*. In contrast, for *E. urophylla* and *E. grandis*, the main drivers were ammonium and nitrate, respectively. Although we do not infer that there is necessarily a causal effect between those correlations, there may still be an unknown reason for them to occur. Therefore, we suggest that *Eucalyptus* species exert a strong effect on soil chemical and microbiological attributes, including soil fungal community structure.

In addition, the cited species-specific correlations suggest that there is a response in terms of higher AMF colonization in *Eucalyptus* species with low and intermediate drought tolerance, especially for *E. urophylla*, whereas high drought-tolerant species correlate less with AMF (*E. brassiana* and *E. camaldulensis*).

## Data Availability Statement

The datasets generated and analysed during the current study are available from the corresponding author on reasonable request.

## Author Contributions

BL: Conceptualization, Formal analysis, Methodology, Roles/Writing - original draft, writing review, and editing. AS: Formal analysis, Roles/Writing - original draft, writing review, and editing. MC, HF, MT, VA, PA, and SS: Methodology, Roles/Writing - original draft, writing review, and editing. AP: Conceptualization, Roles/Writing - original draft, writing review, and editing. JG and EC: Conceptualization, Methodology, Roles/Writing - original draft, writing review, and editing. All authors contributed to the article and approved the submitted version.

## Funding

We thank the São Paulo Research Foundation (FAPESP) - Brazil (2019/13436-8, 2018/20607-0, 2018/20553-8, 2017/16608-9, and 2016/18944-3), France (2018/12665-0), and the National Council for Scientific and Technological Development (CNPq), Brazil (131169/2017-3) for financing this study.

## Conflict of Interest

The authors declare that the research was conducted in the absence of any commercial or financial relationships that could be construed as a potential conflict of interest.

## Publisher’s Note

All claims expressed in this article are solely those of the authors and do not necessarily represent those of their affiliated organizations, or those of the publisher, the editors and the reviewers. Any product that may be evaluated in this article, or claim that may be made by its manufacturer, is not guaranteed or endorsed by the publisher.
