# Supplementary material for: Arbuscular Mycorrhizal Fungi and Soil Quality Indicators in Eucalyptus genotypes With Different Drought Tolerance Levels
Source: Front Fungal Biol. 2022 Jun 20;3:913570. doi: 10.3389/ffunb.2022.913570 (PMC10512215; doi:10.3389/ffunb.2022.913570)
Supplement: Supplementary file 1 [file DataSheet_1.docx]

Supplementary Material

# Supplementary Tables

**Table S1.** Drought tolerance, relative growth, rooting capacity of Eucalyptus species commonly used in Brazilian plantation. Adapted of Gonçalves et al. (2017).

| Species/hybrids | Drought Toleramce | Growth | Rooting |
| --- | --- | --- | --- |
| *Eucalyptus brassiana* | Tolerant | Good | Good |
| *Eucalyptus camaldulensis* | Tolerant | Regular | Very good |
| *Eucalyptus citriodora* | Moderate tolerante | Regular | Regular |
| *Eucalyptus cloeziana* | Moderate tolerante ^*^ | Good | Regular |
| *Eucalyptus grandis* | Low tolerance | Very good | Good |
| *Eucalyptus urophylla* | Low tolerance ^**^ | Good | Very good |

^*^According to Sousa (2000) and Alfenas et al. (2004); ^**^According to Flores et al. (2016)

**Table S2.** Global RDA and forward selection results with bonferroni correction

|  | RDA results | |  |  |  |
| --- | --- | --- | --- | --- | --- |
|  | Df | | Variance | F | p-value |
| Model | 15 | | 0.53279 | 1.6413 | 0.006** |
| Residual | 44 | | 0.95218 |  |  |
| Forward selection results | | | | | |
|  | Df | R^2^ adj | AIC | F | p-value |
| Zn | 1 | 0.075953 | 414.99 | 5.8496 | 0.017 * |
| P | 1 | 0.147742 | 411.09 | 5.8856 | 0.013 * |
| All variables |  | 0.254869 |  |  |  |

Df: degrees of freedom, R^2^ adj: R^2^ adjusted, AIC: Akaike information criteria, **: p<0.01; *: p<0.05

**Table S3**. Multigroup model fit index obtained from the structural equation modeling for each *Eucalyptus* species

|  | Goodness of statistical fit | | | | | | | NMDS |
| --- | --- | --- | --- | --- | --- | --- | --- | --- |
| *Eucalyptus* species | *X*² | df | *p*-value | CFI | TLI | RMSEA | SRMR | Stress value |
| *E. brassiana* | 72.54 | 8 | 0.00 | 1.00 | 1.00 | <0.05 | <0.05 | 9.90E+01 |
| *E. camaldulensis* | 38.21 | 8 | 0.00 | 1.00 | 1.00 | <0.05 | <0.05 | 0.02 |
| *E. citriodora* | 12.69 | 8 | 0.12 | 1.00 | 1.00 | <0.05 | <0.05 | 0.12 |
| *E. cloeziana* | 78.56 | 8 | 0.00 | 1.00 | 1.00 | <0.05 | <0.05 | 0.02 |
| *E. grandis* | 67.79 | 8 | 0.00 | 1.00 | 1.00 | <0.05 | <0.05 | 0.04 |
| *E. urophylla* | 39.54 | 8 | 0.00 | 1.00 | 1.00 | <0.05 | <0.05 | 0.05 |
| Benchmark values^1,2^ | - | - | >0.05 | <0.95 | <0.90 | <0.05 | <0.09 | <0.05 |

*X*²: Chi-square test, df: degrees of freedom, *p*-value: Chi-square significance, CFI: comparative fit index, TLI: Tucker-Lewis Index, RMSEA: root-mean-square error of approximation, SRMR: standardized root mean square residual and stress value obtained in the NMDS: non metric multidimensional scaling, used (first score) to compose the compound variable soil fungal community structure.¹Benchmark values for a good model fit in structural equation modeling according to Fan et al. (2016).^2^Stress value considered good according to Clarke (1993).

**Table S4**. Number of positive and negative interaction considering direct and indirect correlations obtained by the structural equation model of relationships between soil microbiological attributes and the soil fungal community structure for *Eucalyptus* species

|  | Direct correlations with soil fungal structure | | Indirect correlations among soil microbiological attributes | |
| --- | --- | --- | --- | --- |
|  | Positive | Negative | Positive | Negative |
| *E. brassiana* | 4 | 4 | 16 | 12 |
| *E. camaldulensis* | 5 | 3 | 21 | 7 |
| *E. citriodora* | 7 | 1 | 20 | 8 |
| *E. cloeziana* | 5 | 3 | 22 | 6 |
| *E. grandis* | 5 | 3 | 19 | 9 |
| *E. urophylla* | 3 | 5 | 20 | 8 |

# Supplementary Figures

**
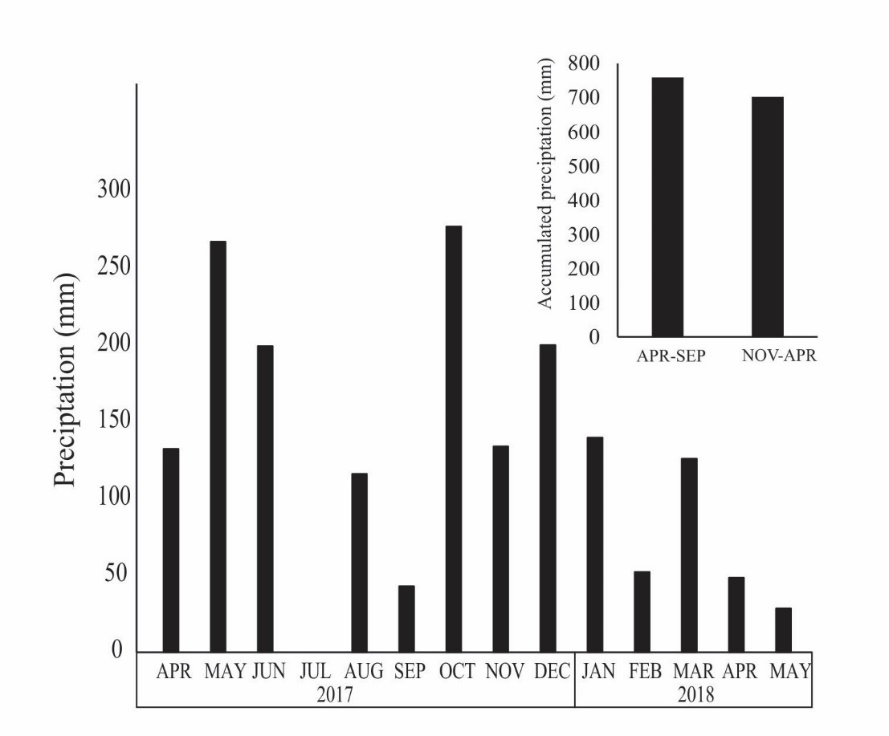
**

**Figure S1**. Monthly rainfall up to six months before the first sampling period (October 2017 and May 2018) and the accumulated rainfall in the 6-month period prior to each sample date (upper right insert).

**
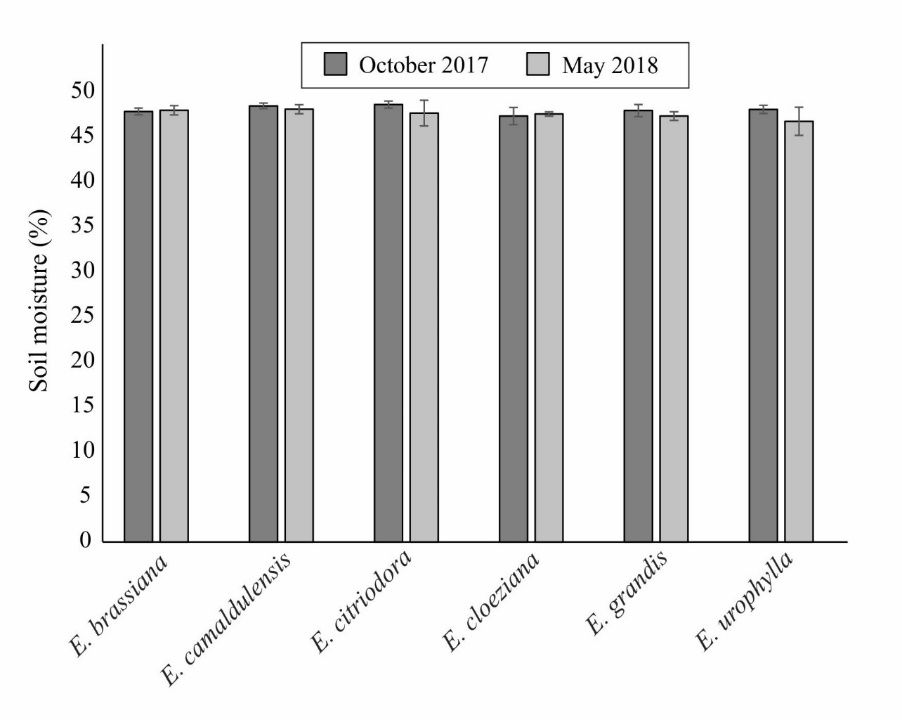
**

**Figure S2**. Soil moisture in the two sampling periods in the different Eucalyptus species.


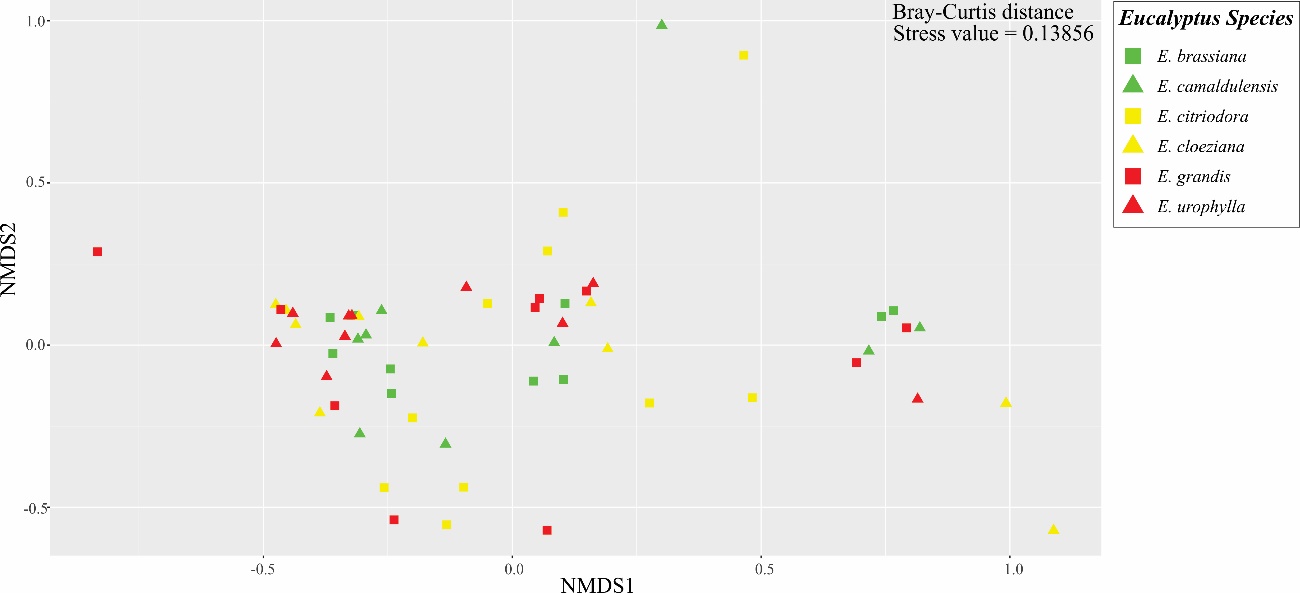


**Figure S3**. Non-metric multidimensional scaling (NMDS) considering the different Eucalyptus species.

# Supplementary References

Alfenas, A. C., Zauza, E. A. V., Mafia, R. G., and Assis, M. T. F. (2004). Clonagem E Doenças do Eucalipto. Ed. M. G. Viçosa, Minas Gerais, Brazil (UFV), 442 p.

Clarke, K., R. (1993). Non-parametric multivariate analyses of changes in community structure. *Austral Ecol*. 18, 117-143. doi: 10.1111/j.1442-9993.1993.tb00438.x

Fan, Y., Chen, J., Shirkey, G., John, R., Wu, S. R., Park, H., et al. (2016). Applications of Structural Equation Modeling (SEM) in Ecological Studies: An Updated Review. Ecol. Process. 5 (1), 1–12. doi: 10.1186/s13717-016-0063-3

Flores, T. B., Alvares, C. A. A., Souza, V. C., and Stape, J. L. (2016). Eucalyptus No Brasil: Zoneamento Climático E Guia Para Identificação (Piracicaba: Instituto de Pesquisas e Estudos Florestais).

Gonçalves, J. L. M., Alvares, C. A., Rocha, J. H. T., Brandani, C. B., and Hakamada, R. (2017). Eucalypt Plantation Management in Regions With Water Stress. South. For. 79 (3), 169–183. doi: 10.2989/20702620.2016.1255415

Sousa, J. S. I. (2000). Enciclopédia Agrıcola Brasileira ́ Vol. Vol. 3 E-H (São Paulo: da Universidade de São Paulo), 510p.
